# ENT manifestations of tuberculosis: an important aspect of ENT practice

**DOI:** 10.11604/pamj.2020.36.295.24823

**Published:** 2020-08-17

**Authors:** Shilpam Sharma, Amit Kumar Rana

**Affiliations:** 1 Department of ENT, Vardhaman Mahaveer Medical College and Safdarjung Hospital, New Delhi, India,; 2 Department of Otorhinolaryngology and Head Neck Surgery, SRMS Institute of Medical Sciences, Bareilly (UP), India

**Keywords:** Extra-pulmonary, lymphadenitis, oral tuberculosis

## Abstract

Tuberculosis involving organs other than the lungs is termed as 'extra pulmonary tuberculosis'. Tuberculosis (TB) remains a worldwide public health problem despite the fact that the causative organism was discovered more than 100 years ago. The present study was conducted to assess different manifestations of tuberculosis affecting the ear, nose and throat (ENT) in patients attending the outpatient department in a total of 520 cases of tuberculosis. One hundred and eight cases were of extra pulmonary tuberculosis. Sixty nine cases had the manifestations of TB in the ENT region. These included patients with tuberculous cervical lymphadenopathy (91.35), laryngeal TB (4.3%), tuberculous otitis media (1.4%), nasal TB (1.4%) and oral tuberculosis (1.4%). Extra pulmonary tuberculosis constitutes about 15-20% of all tuberculosis cases as per WHO survey and it is 20.6% in the present study.

## Introduction

Tuberculosis (TB) is a chronic granulomatous, infectious and communicable disease caused by *Mycobacterium tuberculosis* [1]. Tuberculosis usually attacks the lungs but can also affect other parts of the body. Tuberculosis involving organs other than the lungs is termed as 'extra pulmonary tuberculosis'. Tuberculosis remains a worldwide public health problem despite the fact that the causative organism was discovered more than 100 years ago and highly effective drugs are available for preventing and curing the disease. According to the estimates, there are 15-20 million cases of infectious tuberculosis in the world. Globally in 2012 an estimated 8.6 million people developed tuberculosis and 1.3 million died from the disease [2]. This pool of tuberculosis is maintained by the occurrence of 7.25 million new cases annually [3]. Out of the extra pulmonary manifestations of tuberculosis, ear, nose and throat manifestations are mainly in the form of cervical lymphadenopathy, otitis media, laryngitis, pharyngitis and nasal TB [4]. The present study was conducted to assess different manifestations of tuberculosis affecting the ear, nose and throat in patients attending the outpatient department of a tertiary care hospital in Western Uttar Pradesh.

## Methods

This prospective study was conducted in Department of Otorhinolaryngology and Head Neck Surgery of a tertiary care center of Uttar Pradesh, India. All cases diagnosed with extra pulmonary tuberculosis in ear, nose and throat region of all age group attending ENT OPD and willing to be part of study were included in study. A written consent was obtained from patients. The study was conducted after taking permission from the institutional ethics committee. The time period of this study was January 2018 to December 2019. A detailed ENT history was obtained from all the patients in order to assess the involvement of the ear, nose and throat. Details regarding demographic data and presenting complaints. Emphasis was placed especially on symptoms like chronic ear discharge, hemoptysis, change in voice, chronic cough, persistent neck swellings, fever and weight loss. Relevant past and family history of tuberculosis was also obtained. General, systemic and complete ENT examination was carried out. All the patients were subjected to X-ray chest posteroanterior (PA) view. Radiological examination of the soft tissue neck cervical spine and X-ray Schuler´s view for mastoid were carried out. Endoscopic examination including otoendoscopy, diagnostic nasal endoscopy and direct laryngoscopy was performed wherever indicated. Ultrasound neck and fine needle aspiration cytology (FNAC) was performed on all suspected neck swellings. Investigations also included culture and sensitivity and AFB staining of the sputum, pus from discharging sinuses, laryngeal secretions and ear discharge. Direct laryngoscopic and lymph node biopsy was done if required for suspected laryngeal lesions. All data were collected, tabulated and analyzed.

## Results

A total of 520 cases of tuberculosis diagnosed in our institute during the period of review, 108 cases were of extra pulmonary tuberculosis (EPTB) either in isolation or associated with concomitant pulmonary tuberculosis (PTB). Of the 108 patients with EPTB, 69 cases had the manifestations of TB in the ENT region. These included patients with tuberculous cervical lymphadenopathy, laryngeal TB, tuberculous otitis media (TBOM), nasal TB and oral tuberculosis (Table 1).

**Table 1:** nature of lesion

Nature of lesion	No. of patients	%
Tubercular lymphadenitis	63	91.3%
Tubercular otitis media	1	1.4%
Laryngeal tuberculosis	3	4.3%
Nasal tuberculosis	1	1.4%
Oral tuberculosis	1	1.4%
Total	69	100%

**Figure 1:**
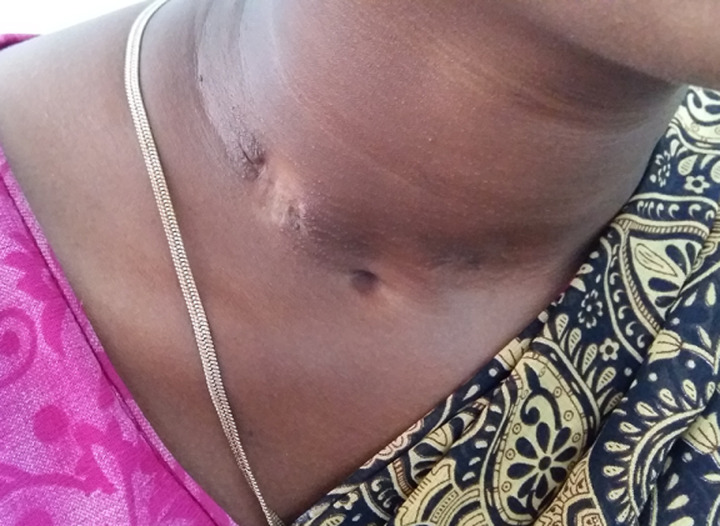
tuberculous sinus in neck

**Tubercular lymphadenitis:** the commonest presentation of extra-pulmonary tuberculosis in ENT region was cervical tuberculous lymphadenopathy. There were 35 males and 28 females. The commonest age group affected was the third decade of life and patients came with complaint of neck swelling. There were other complaints like cough with expectoration (22 cases), fever (18 cases) and discharging sinus (1 case) (Figure 1). There were multiple matted lymph nodes in 60 cases and single lymph node in 3 cases. Bilateral lymph node involvement was noted in 39 cases. In majority of the cases lymph nodes in the anterior triangle were involved. The next common group of lymph nodes involved were the posterior triangle. The diagnosis was confirmed by USG neck and FNAC of the neck nodes. FNAC diagnosis was in the form of granulomatous lymphadenopathy or chronic lymphadenitis consistent with the findings of tuberculosis. Thirty three patients had pulmonary tuberculosis too. The patients were started on category I anti tuberculous treatment (ATT) according to Revised National Tuberculosis Control Programme (RNTCP) for 6 months. They were kept on monthly follow up till the completion of treatment and as per requirements after that. In 55 cases the swelling subsided by the end of the treatment course. In 8 patients the swelling remained of same size in spite of taking full treatment (Figure 1).

**Tubercular otitis media:** out of total 108 cases of extra pulmonary tuberculosis, one case was identified as having tubercular otitis media. The presenting symptoms were persistent ear discharge not responding to antibiotics, severe to profound hearing loss disproportionate to hearing loss and facial palsy. The complaint of the patient was recurrent ear discharge, profound hearing loss with infra nuclear facial palsy. On examination, large tympanic membrane perforation was seen with multiple pale granulations in middle ear. On culture and sensitivity of discharge, *Mycobacterium tuberculosis* were seen. The patient underwent modified radical mastoidectomy and the granulation tissue was sent for histopathological examination and diagnosis of tubercular otitis media was made. The patient was put on antitubercular treatment.

**Laryngeal tuberculosis:** the common presenting symptoms of laryngeal tuberculosis were hoarseness and odynophagia, along with constitutional symptoms of tuberculosis. Total 3 cases were diagnosed to have laryngeal tuberculosis. The patients presented with cough with expectoration and hoarseness. On laryngeal examination polypoidal changes were seen in interaytenoid region along with mouse nibbled epiglottis in 1 case and congestion was seen in the vocal cords in 2 cases. Stripping was done and specimen was sent for histopathology examination. Diagnosis of laryngeal tuberculosis was made. Patient was started on antitubercular treatment. All these patients were sputum positive, however typical signs of laryngeal tuberculosis were not seen.

**Nasal tuberculosis:** nasal obstruction and blood stained nasal discharge are the most common presenting symptom of nasal tuberculosis. One case of nasal tuberculosis was reported during the study period. The patient complained of persistent nasal discharge and nasal obstruction. On examination a pale polypoidal mass was seen in the left nasal cavity. The patient underwent functional endoscopic sinus surgery and the mass was removed which was then sent for histopathological examination which confirmed the diagnosis of nasal tuberculosis. The patient was put on ATT and got relieved of symptoms. This case was secondary to pulmonary tuberculosis, though cases of primary tuberculosis have also been reported.

**Figure 2:**
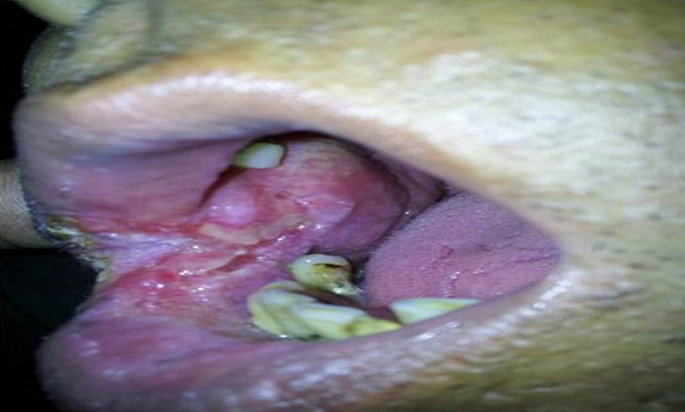
tuberculous ulcer

**Figure 3:**
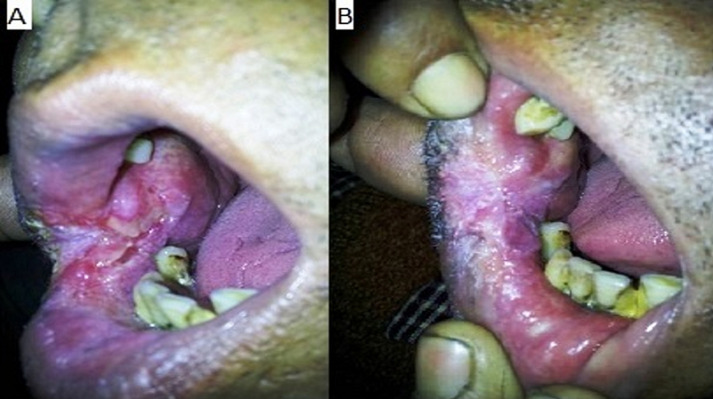
A) before treatment of the tuberculous ulcer; B) after treatment

**Oral tuberculosis:** patient came with the chief complaint of difficulty in opening mouth and gradually increasing painless ulcer on the buccal mucosa (Figure 2). On clinical examination crusts were present on angle of mouth with an ulcer with well-defined rolled up margins. The base was indurated, granular and non tender and did not bleed on touch. Oral hygiene was poor. The blood investigations were within normal limits except erythrocyte sedimentation rate (ESR) which was 45mm. X-ray chest revealed ill-defined opacities in both upper zones suggestive of pulmonary Koch´s. Sputum for AFB was negative. The patient underwent biopsy under local anesthesia. Histopathological report revealed squamous epithelium with features of hyperplasia. Subepithelial tissue showed granulomatous pathology consisting of epitheloid cells and multinucleated Langhan´s type of giant cells and areas of caseation (Figure 3). Acid fast bacilli were identified on Ziehl-Neelsen´s staining. Features were suggestive of tuberculous pathology. Patient was treated as a new case of TB and DOTS category 1 regimen was started. Significant improvement was seen within 15 days of starting the treatment, in the form of decrease in the size and erythema of the ulcer (Figure 3).

## Discussion

Tuberculosis is a global disease and it is estimated that extrapulmonary tuberculosis constitutes 15 to 20 per cent of tuberculosis cases in general practice among HIV-negative adults in India [5]. In our study 520 cases of tuberculosis were evaluated out of which 108 cases were of extra pulmonary type. In our study cervical TB lymphadenitis accounted for 95.5% of cases of extra pulmonary tuberculosis in the ENT region. In the present study the pattern of lymph node involvement showed multiple lymph node group involvement in 96% of the cases and the lymph nodes of the posterior triangle were the most common lymph node involved (78%). This corresponds to the findings in the study of Bayazit Ya *et al*. [6]. FNAC was the diagnostic investigation for the lymph node tuberculosis except in 2 cases in which lymph node biopsy was done. FNAC confirmed the diagnosis in most of the cases which is in accordance with the study by Chakravorty S *et al*. [7]. Tubercular otitis media is a rare manifestation of tuberculosis [8]. It accounted for 1.5% of EPTB case in the present study. In our study, the case with tubercular otitis media had a finding of recurrent ear discharge, not responding to usual antibiotics, hearing loss with infranuclear facial palsy. On examination, a large tympanic membrane perforation was seen.

On culture and sensitivity of discharge, *Mycobacterium tuberculosis* were seen. The classical multiple perforations were not noted. Histopathology report (HPE) of diseased tissue from the ear is the surest way to confirm the diagnosis of TBOM. This has also been reported by other studies [8,9]. Dysphonia is the commonest presenting complaint with pain also being a prominent feature in laryngeal TB [4,10,11]. Our patients complained of hoarseness. It is believed that the recent resurgence in reported cases of LTB is due to increase in HIV cases [10-12]. There was one case in our study. A direct laryngoscopy is necessary not only to confirm diagnosis and rule out malignancy but also to take tissue for HPE [10,11]. Nasal TB is a very rare entity even in countries with high disease load [12]. We had only one case over a period of study. Our patient was a 21 year old female. The complaint of blood stained nasal discharge reported by our case was also noted by Dixit *et al*. [13]. The case in the present study had nasal mass with sinus involvement. However, the commonest feature of nasal tuberculosis is septal involvement with perforation resulting in external nasal deformity. A high index of suspicion is the only key especially since there can be varied differential diagnosis [14].

The typical lesion of oral TB is an irregular, superficial or deep, painful ulcer which tends to increase slowly in size. It is frequently found in areas of trauma and may be mistaken clinically for a simple traumatic ulcer or even carcinoma. The present case there was an irregular, superficial painless ulcer. It appears most likely that the organisms are carried in the sputum and enter the mucosal tissue through a break in the surface, or hematogenous route, deposited in the submucosa and subsequently proliferate and ulcerate the overlying mucosa. In the present case the patient was sputum negative hence the route of infection appears to be hematogenous in nature. The patient had poor oral hygiene which could also facilitate the infective process. It is suggested that when granulomatous inflammation is confirmed by tissue biopsy, TB should also be one of the differential diagnosis, especially in countries that still have higher TB incidence [15]. According to WHO global tuberculosis report 2013 diagnosis of extra pulmonary tuberculosis should be based on one culture-positive specimen, or histological or strong clinical evidence consistent with active extra pulmonary disease, followed by a decision by a clinician to treat with a full course of anti-TB chemotherapy. A patient in whom both pulmonary and extra pulmonary TB has been diagnosed should be classified as a pulmonary case [2].

## Conclusion

Extra pulmonary tuberculosis constitutes about 15-20% of all tuberculosis cases as per WHO survey and it is 20.6% in the present study. Although incidence of tuberculosis is on the decline in developed countries, but still pulmonary and extra pulmonary tuberculosis cases do exist. Even when ENT manifestations of tuberculosis have reduced due to health awareness, early detection and treatment, yet tuberculosis should be considered as a differential diagnosis in case of chronic lymphadenopathy, chronic discharging ears, hoarseness, nasal masses with blood stained discharge and other chronic long standing ENT diseases. Changing patterns of presentations of laryngeal, aural and nasal tuberculosis was observed in these cases.

### What is known about this topic

- Tuberculosis has reemerged as a disease in the last two decades;
- Now extra-pulmonary manifestations have taken the center stage in tubercular presentations;
- Most commonly, cervical lymphadenopathy is the presenting complaint in patients.

### What this study adds

- Our study highlights more common extra pulmonary presentations in Indian scenario which is endemic to tuberculosis;
- Our study emphasizes on reporting oral tuberculosis as an important finding not commonly seen elsewhere;
- In ENT practice, a recurrent otitis media not responding to medical or surgical management should arise suspicion of tubercular otitis media and a trial of anti- tubercular treatment gives good response.
